# 

**Published:** 1916-05-13

**Authors:** 


### PASSING EVENTS AND TOPICS.

### The Royal Free Hospital Appeal.

The Lord Mayor will preside at a meeting at the Mansion House on May 26, at 3.30 p.m., in support of the Royal Free Hospital's appeal for funds to extend its work so as to provide for the welfare of infant life and for the general purposes of the hospital.

### Medical Casualties in Ireland.

The casualties suffered by our Army in suppressing the Irish rebellion include two officers of the Royal Army Medical Corps—namely, Captain J. T. McCullagh, who is wounded, and Lieutenant and Quartermaster E. H. Atkins, who is reported missing.

### The Loss of H.M.S. "Russell."

The list of casualties resulting from the sinking of H.M.S. *Russell* contains the names of two surgeons, both of whom died of wounds on April 28—namely, Fleet-Surgeon W. R. Center, R.N., and Surgeon Philip D. Pickles, R.N.V.R.

### Longford Hall as a Hospital.

The military authorities are increasing the accommodation for wounded soldiers in Manchester, and this week Longford Hall—a fine mansion standing in a public park at Stretford—will be added to the list of hospitals. Longford Hall Hospital has 100 beds.

### American Red Cross Work in Germany.

According to the Washington correspondent of the *Times*, the question of the shipment by the American Red Cross of hospital supplies to Germany is again under consideration. It is understood that in default of permits to pass the blockade the American Red Cross is obliged to notify its branches that no more supplies can be received for Germany. The opinion held in the United States is that Great Britain will refuse to allow shipments unless the American Red Cross should establish units in Germany, in which case supplies would be allowed to reach these units.

### The Book Market.

LIST OF NEW BOOKS RECEIVED FROM THE FOLLOWING PUBLISHERS:—

Cambridge University Press: "Occupations, from the Social, Hygienic, and Medical Points of View." By Sir Thomas Oliver, M.A., M.D., LL.D., D.Sc. Price 6s. net.

Cassell and Co.: "Serums, Vaccines, and Toxins in Treatment and Diagnosis." By W. C. Bosanquet, M.A., M.D., and John W. H. Eyre, M.D., M.S. Third Edition. Price 9s. net.

H. K. Lewis and Co., Ltd.: "The Pathology of Tumours." By E. H. Kettle, M.D., B.S. Price 10s. 6d. net. "The Diagnosis and Treatment of Heart Disease." By E. M. Brockbank, M.D. Second Edition. Price 3s. 6d. net.

Percy Lund, Humphries and Co., Ltd.: "Surgical Bandages and Dressings." By Alice Scott. Price 1s. net.

G. P. Putnam's Sons: "The Case of Edith Cavell." By James M. Beck. Price 6d. net.

### Institutional and Public Reports.

"Feeble-Minded in Ontario." Tenth Report, for the year ending October 31, 1915. Printed by order of the Legislative Assembly of Ontario.

In addition to those specially noticed, annual reports have been received from the following: Board of Health of the Town of Montclair, New Jersey, 1915; Transactions of the Incorporated Sanitary Association of Scotland, 1915; Bradford Health Committee, Infants' Department, Report of Chief Medical Officer.

### Obituary.

The death is reported of Dr. F. C. P. Howes, of Lincoln, who was at one time resident medical officer at the Eastern Dispensary, Bath, and had held resident appointments at Birmingham and Lincoln. He was a son of the late Rev. T. G. F. Howes, Rector of Belton, Suffolk, and received his medical training at King's College Hospital, London, and at Edinburgh University, taking the M.D. degree at Edinburgh in 1864.

### HOSPITAL RESULTS IN 1915.

### More Early Reports of the Voluntary Hospitals.

ANCOATS HOSPITAL, MANCHESTER.—There is an appreciable fall in the numbers of in-patients, out-patients, and casualties, but the average stay in the wards increased from sixteen to twenty days; 165 wounded and sick soldiers were received at the hospital and 325 at the Warford Convalescent Home, where the accommodation has been increased. Income was £11,691 and expenditure £11,955, and the debt to bankers now stands at £2,246.

BECKETT HOSPITAL AND DISPENSARY, BARNSLEY.—Three hundred and forty wounded and sick soldiers have been treated in the wards during 1915. To help with the increased expenditure the Saturday and Sunday Committees put forth a special effort and raised £3,087, as against £1,984 in 1915; by means of outdoor musical entertainments £739 was contributed to this fund.

CENTRAL LONDON OPHTHALMIC HOSPITAL.—The number of patients largely increased last year. Six out of ten of the surgical staff have joined the Forces, as well as many clinical assistants, house surgeons, and members of the nursing and general staff. The total ordinary income was £3,261 and the expenditure £2,684. This prosperity is evidence that the sound policy of moving the work to a modern building, properly equipped, is appreciated by the giving public.

EVERSFIELD CHEST HOSPITAL, ST. LEONARDS-ON-SEA.—The thirty-first annual report states that although annual subscriptions show an appreciable decrease the amount received in donations is in excess of the previous year. The hospital is supported by the Corporation of the City of London (the Lord Mayor is the president), King Edward's Hospital Fund, the King Edward VII. Welsh National Memorial Association, and the Metropolitan Hospital Saturday Fund. In consequence of the landslip of January 1912 the institution has been put to considerable expense in reconstructing a portion of the cliff wall. An exact description is given of the status, age, etc., of each of the 192 patients discharged, showing condition on discharge, gain or loss of weight, etc. The report is very well arranged and is most interesting.

GRAVESEND HOSPITAL.—The front cover of the report for 1915 has upon it a picture of an alert-looking soldier saluting and saying "Thank you," with a view of the hospital as a background. This soldier represents the 381 military cases who were treated in the emergency war beds and the 246 who found the out-patient department serviceable, as well as the civilians who, being ineligible for the Army in consequence of some physical defect, were rendered fit for enlistment by treatment at the hospital. The ordinary income for the year was £4,848 (including £1,887 patients' contributions), and the ordinary expenditure £5,387. Mr. Walter Pearson, the secretary, is serving with the R.A.M.C.

HOSPITAL FOR CONSUMPTION, BROMPTON.—Facilities offered to the authorities for the admission of sailors and soldiers discharged from the Services suffering from consumption continue to be made full use of. An appeal issued in April 1915 resulted in donations amounting to over £9,000 being received. On the expenditure side it is shown that provisions alone caused an additional cost of £3,000. The total income of the hospital and Frimley Sanatorium was £46,305, and the expenditure £45,318. In view of the increased cost of production, only a limited number of copies of the report have been printed, and donors and subscribers have been asked to say if it is desired that a copy shall be sent as usual. In view of the fact that a large proportion of the expense of production has to be incurred in any case, it would be interesting to see actual figures illustrating the suggested economy. The propaganda value of an annual report is undoubtedly a consideration not to be lightly dispensed with.

MONTGOMERY COUNTY INFIRMARY.—The demand on the in-patient department being so abnormal during 1915, the usual number of beds had to be increased. Two hundred and eighty-five patients were received, including fifty-three sick and wounded soldiers. Surgery and dispensary expenditure increased from an average of 4s. 10d. per patient in the three previous years to 6s. 6d. What are described as "housekeeping expenses" (the accounts are not on the Uniform System) amounted to 9s. 1d. per patient, a decrease of nearly 4d. on the previous year. There is a slight deficit on the year's working.

ST. BARTHOLOMEW'S HOSPITAL, LONDON.—In a note under this heading last week we invited an explanation of a fall in the annual subscriptions from £5,386 in 1914 to £3,153 in 1915. Through the courtesy of Mr. Hayes we are able to state that the difference is represented by a loss to the hospital from the expiration of subscriptions promised by governors for the three years 1912-14 inclusive, amounting in 1915 to £2,742 3s. We are glad to note from a closer examination of the subscriptions and donations for the years 1914 and 1915 that the active appeal work done in 1915 raised the sum received from donations from £4,849 to £9,948, an increase of £5,099, to which must be added a further £3,000, being grants obtained from executors by special application, including the sum of £2,000 from the trustees of the late Sir William Dunn, Bart. Another interesting feature which shows the good financial work now being done is that whereas the number of contributors in 1914 was 1,294, they had increased in 1915 to 2,057. Our hearty congratulations to Lord Sandhurst and Mr. Hayes on these results.

ST. BARTHOLOMEW'S HOSPITAL, ROCHESTER, founded by Gundulf, Bishop of Rochester, in 1078.—The report claims that the requirements of the civil population have been met, but that two wards, which dealt with 449 cases in 1915, have been set aside for wounded and sick soldiers. There was a balance of £2,028 to the good on last year's accounts. A flourishing Linen League, founded five years ago, has continued its successful work.

ROYAL EYE HOSPITAL, SOUTHWARK.—The annual report has a feature we do not remember to have seen elsewhere; after each of the names of the honorary medical staff is given a list of other special hospital appointments held. Last year fifty-three sailors and soldiers received treatment in the wards and a large number attended the out-patient department. The ordinary income increased from £3,470 to £4,691, and the expenditure from £4,982 to £5,376. Legacies amounting to £2,541 were received. Mr. Conrad Thies, late secretary of the Royal Free Hospital, has been acting as honorary secretary, and has now been relieved by Mr. Charles H. Warren.

RATES OF SUBSCRIPTION: Twelve months, 6/6; Six months, 4/-; Three months, 2/-, post free to any address in the United Kingdom. Abroad, Twelve months, 8/8; Six months, 5/-; Three months, 2/6, post free.—Address The Subscription Dept., "THE HOSPITAL," The Hospital Building, 28/29 Southampton Street, Strand, London, W.C.

